# Pancreatic Islet Cell Hormones: Secretion, Function, and Diabetes Therapy

**DOI:** 10.1002/mco2.70359

**Published:** 2025-09-06

**Authors:** Jinfang Ma, Mao Li, Lingxiao Yang, Qingxing Xie, Rongping Fan, Xi Lu, Xing Huang, Nanwei Tong, Zhenyu Duan

**Affiliations:** ^1^ Department of Endocrinology and Metabolism, Center for Diabetes and Metabolism Research, Division of Pancreatic Surgery, Department of General Surgery, Department of Radiology, Huaxi MR Research Center (HMRRC), Institution of Radiology and Medical Imaging, West China Hospital Sichuan University Chengdu China

**Keywords:** diabetes, hormonal crosstalk, hormone secretion, pancreatic islet, therapeutic innovation

## Abstract

The pancreatic islets of Langerhans, which are composed of α, β, δ, ε, and PP cells, orchestrate systemic glucose homeostasis through tightly regulated hormone secretion. Although the precise mechanisms involving β cells in the onset and progression of diabetes have been elucidated and insulin replacement therapy remains the primary treatment modality, the regulatory processes, functions, and specific roles of other pancreatic islet hormones in diabetes continue to be the subject of ongoing investigation. At present, a comprehensive review of the secretion and regulation of pancreatic islet cell hormones as well as the related mechanisms of diabetes is lacking. This review synthesizes current knowledge on the secretion mechanisms of insulin, glucagon, somatostatin, ghrelin, and pancreatic polypeptides, emphasizing their functional crosstalk in diabetes. Emerging advances include CRISPR‐based β‐cell regeneration, bioengineered islet transplantation, and bioelectronic interventions aimed at restoring pancreatic function. Future research directions highlight artificial intelligence‐guided prediction of hormone dynamics, therapeutics targeting the gut microbiome–islet axis, and tissue‐engineered artificial islets. By integrating mechanistic insights, physiological roles, and translational innovations, this review outlines precision strategies for targeting islet hormone networks, offering a roadmap toward restoring metabolic equilibrium in diabetes.

## Graphical abstract

The pancreatic islets, composed of α, β, δ, ε, and PP cells, play a central role in maintaining glucose homeostasis. This review examines the mechanisms of islet hormone secretion and their functional crosstalk networks, alongside the pathophysiology of diabetes driven by hormonal dysregulation. Furthermore, we discuss current and future therapeutic strategies targeting islet hormones for diabetes treatment.

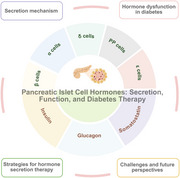

## 1 Introduction

The pancreatic islets, also known as islets of Langerhans, are clusters of cells dispersed throughout the pancreas that play a pivotal role in maintaining glucose homeostasis [1]. These islets contain several types of endocrine cells, each of which secrete distinct hormones crucial for metabolic regulation: α cells produce glucagon, β cells secrete insulin, δ cells release somatostatin, ε cells generate ghrelin, and PP cells produce a pancreatic polypeptide (PP) [2, 3]. The distribution of these cell types within the islets varies across species, with β cells comprising approximately 60–80% of the total islet mass, making them the most abundant cell type [4, 5, 6]. They are located predominantly at the core of mouse islets and are surrounded by α cells, which account for approximately 30–40% of the islet population [7, 8]. δ cells, ε cells, and PP cells make up the remaining fraction, strategically positioned to modulate the activity of the other cell types. This spatial arrangement facilitates paracrine interactions, enabling the secretion of one hormone to influence the function of adjacent cells, thereby enhancing the efficiency of glucose metabolism regulation [9, 10].

Insulin, secreted by β cells, acts as an anabolic hormone that promotes the uptake of glucose into tissues such as muscle and fat while inhibiting hepatic glucose production [11, 12]. In contrast, glucagon, released by α cells, serves as a catabolic hormone, stimulating glycogenolysis and gluconeogenesis to increase blood glucose levels during fasting states [13, 14]. Somatostatin, produced by δ cells, exerts an inhibitory effect on both insulin and glucagon secretion, helping to maintain stable glucose concentrations [15]. Ghrelin, although primarily produced by the stomach, has been identified in pancreatic ε cells and plays a role in appetite regulation [16]. PP, which is secreted by PP cells, influences gastrointestinal motility and pancreatic hormone secretion [17]. The intricate balance between these hormones is critical for maintaining metabolic homeostasis. Insulin and glucagon act in opposition to each other, forming a feedback loop that adjusts blood glucose levels in response to dietary intake and energy demands [18, 19, 20]. Disruptions in this delicate equilibrium can lead to metabolic disorders, including diabetes mellitus [21].

Diabetes mellitus refers to a group of metabolic diseases characterized by chronic hyperglycemia resulting from defects in the secretion or action of hormones produced by the pancreatic islets [22]. According to recent epidemiological data in China, an estimated 233 million individuals were living with diabetes in 2023, and the age‐standardized rate of prevalence for diabetes in China will continue to increase linearly over time, reaching 29.10% in 2050, assuming that the existing trends are persistent [23]. Type 1 diabetes (T1D), an autoimmune condition in which β cells are destroyed, leads to absolute insulin deficiency [24]. Type 2 diabetes (T2D), which is more common and often associated with obesity, involves progressive β‐cell dysfunction alongside peripheral insulin resistance [25]. In T2D, early stages are characterized by increased insulin secretion to compensate for insulin resistance; however, over time, β cells fail to sustain this elevated output, leading to relative insulin deficiency [25, 26]. Concurrently, the dysregulation of other islet hormones exacerbates the disease process. For example, inappropriate glucagon secretion contributes to hyperglycemia, whereas impaired somatostatin signaling fails to adequately suppress glucagon and insulin secretion [15, 27]. Moreover, alterations in ghrelin and PP levels may affect appetite regulation and gastrointestinal functions, indirectly impacting glucose metabolism [28, 29].

**FIGURE 1:**
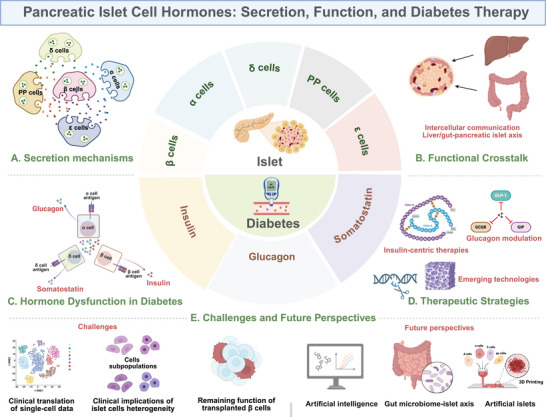
Schematic illustration of pancreatic islet cell hormones related to secretion function and diabetes therapy. (A) Secretion mechanisms of islet hormones in β cells, α cells, δ cells, ε cells, and PP cells. (B) Functional crosstalk of intraislet communication and interorgan signaling in maintaining glucose homeostasis. (C) Aberrant dysfunction of insulin, glucagon, and somatostatin secretion in diabetes. (D) Therapeutic strategies targeting islet hormones, including insulin‐centric therapies, glucagon modulation, and emerging technologies. (E) Challenges and future perspectives for diabetes therapy. Created with Biorender.com. PP, pancreatic polypeptide; GLP‐1, glucagon‐like peptide‐1; GCGR: glucagon receptor; GIP, glucose‐dependent insulinotropic polypeptide.

The secretion and function of pancreatic islet hormones are fundamental to glucose regulation. Understanding the delicate secretion regulation of islet cell hormones and the complex interplay among islet hormones not only sheds light on the pathophysiology of diabetes but also informs therapeutic strategies aimed at restoring hormonal balance. This review delves into the mechanisms of islet hormone secretion, regulatory networks, physiological functions, and current and emerging therapeutic strategies targeting these pathways in diabetes management (Figure 1). By synthesizing recent findings and insights, we aim to provide a comprehensive overview of the field and highlight areas for future investigations.

## 2 Islet Cell Hormones: Secretion Mechanisms

The pancreatic islet orchestrates systemic metabolic homeostasis through the precisely regulated secretion of hormones from its specialized endocrine cells. This section systematically examines the molecular mechanisms governing hormone release from each islet cell type, with particular emphasis on the well‐characterized pathways of insulin (β cells) and glucagon (α cells), while also addressing the less understood secretion dynamics of somatostatin (δ cells), ghrelin (ε cells), and pancreatic polypeptide (PP cells).

### 2.1 Insulin (β Cells)

**FIGURE 2:**
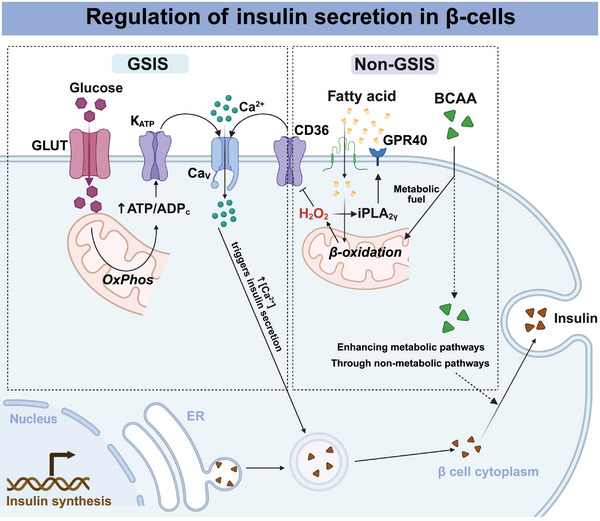
Regulation of insulin secretion in β cells. Classical GSIS: Glucose increases the cellular ATP/ADP ratio, which closes KATP channels and depolarizes the cell. Then, the activation of voltage‐dependent Ca^2+^ channels increases Ca^2+^ entry and stimulates insulin release. Non‐GSIS: Fatty acids and BCAA facilitate insulin secretion via both metabolic and nonmetabolic pathways. Created with BioRender.com. GSIS, glucose‐stimulated insulin secretion; BCAA, branched‐chain amino acids; GLUT, glucose transporter; ER, endoplasmic reticulum; iPLA_2γ_, calcium‐independent phospholipase A_2γ_.

Compared with those of other islet hormones, the mechanisms regulating insulin secretion are well understood. This section discusses the classical GSIS mechanism and emerging research in this area (Figure 2). Additionally, the roles of fatty acids (FAs) and amino acids in modulating insulin release will also be explored.

#### 2.1.1 Glucose‐Stimulated Insulin Secretion

Insulin is primarily secreted in response to elevated glucose concentrations in the blood following a meal; when β cells are exposed to stimulatory glucose, both a “triggering” and an “amplifying” pathway are involved [30], which is also a classic glucose‐sensitive insulin release mechanism. Briefly, the activation of the triggering pathway is initiated by biochemical signals identified over 40 years ago, which involve glucose metabolism to produce ATP, and then the shuttling of ATP‐sensitive potassium (KATP) channels, which are composed of Kir6.2 and sulfonylurea receptor 1 (SUR1) subunits, leads to membrane depolarization and subsequently activates voltage‐gated Ca^2+^ channels [31]. The sharp increase in intracellular Ca^2+^ levels triggers the exocytosis of insulin secretory granules, which release insulin to the cell exterior. The amplifying pathway is triggered even when intracellular Ca^2+^ levels are at their peak, primarily through mechanisms that do not involve KATP channels. During the amplifying phase, primed granules are released, and granules from an internal storage pool are also recruited to the cell surface. In the progression of classical GSIS, glucose is absorbed into β cells through glucose transporter 1 (GLUT1) in humans and through GLUT2 in rodents, but this process is not rate limiting for GSIS. Glucokinase regulates the rate of glucose metabolism in β cells by controlling glucose entry into the glycolytic pathway, leading to its oxidation through the tricarboxylic acid cycle and the production of ATP. An increase in the ATP‐to‐ADP ratio after glucose metabolism results in the closure of KATP channels, depolarization of the membrane, and opening of voltage‐gated calcium channels, which triggers the release of insulin.

The emerging regulatory mechanisms associated with this classical pathway, such as PI (4,5) P2, which regulates the opening of KATP channels, are the subject of ongoing investigation [32]. In theory, loss of KATP channel function causes hyperexcitability, insulin hypersecretion, and even congenital hyperinsulinism, but some cases evolve into insufficient secretion and even diabetes. York et al. [33] reported that increasing extracellular Ca^2+^ beyond normal levels elevates intracellular Ca^2+^ and enhances insulin secretion from sur1 knockout islets to match that of wild‐type islets, which is related to the expression of Trpm5. Another study revealed that TMEM63B, a stretch‐activated cation channel, regulated insulin secretion during the progression of GSIS [34]. Mechanistically, high levels of glucose cause cells to swell and activate TMEM63B, leading to the depolarization of β cells and the secretion of insulin. Consequently, GSIS encompasses intricate regulatory mechanisms that necessitate further investigation for comprehensive elucidation.

The role of mitochondria in insulin release in pancreatic β cells is also constantly being explored [35, 36]. By serving as metabolic and redox centers, mitochondria establish various connections with plasma membrane channels, insulin granule vesicles, the cellular redox balance, and the levels of NADH, NADPH, and calcium homeostasis, impacting insulin secretion [35]. In addition to generating the ATP needed for GSIS, mitochondrial redox signaling affects the transport of Ca^2+^ across the inner mitochondrial membrane [37, 38]. Mitochondrial Ca^2+^ is involved in the initial phase of GSIS and its enhancement by glucagon‐like peptide‐1 (GLP‐1), whereas a rapid increase in glucose levels in primary β cells triggers simultaneous CaV‐dependent [Ca^2+^]c oscillations, causing postponed steady‐state increases in mitochondrial [Ca^2+^]m until they reach saturation [39, 40, 41, 42]. In addition, the metabolic homeostasis of the mitochondria in pancreatic β cells is crucial for insulin secretion. For example, NADPH has traditionally been viewed as a promoter of GSIS [43, 44]. Upon GSIS activation, redox shuttling limits the production of maximum NADH in the mitochondrial matrix, leading to increased NADPH production in the cell cytosol, which signifies the transfer of redox equivalents from the matrix to the cytosol.

In addition, the number of lysosomes and lysosomal enzyme activity are also associated with insulin secretion. Speidel D reported that CAPS1 and CAPS2 regulate insulin content and granule number [45]. Interestingly, CAPS1 and CAPS2 accelerate insulin granule degradation mostly by disrupting the functionality of the lysosomal system. Consequently, the maintenance of lysosomal system homeostasis is integral to the regulation of the GSIS process.

With the development of molecular biological techniques and single‐cell analysis, the heterogeneity of β cells has been increasingly elucidated, encompassing aspects such as biomarkers and molecular regulatory mechanisms. For example, Yu et al. [46] reported a novel means, differential DNA methylation at the imprinted gene *Nnat*, to represent β‐cell heterogeneity established during development. Another study revealed that approximately 40% of islet β cells are involved in 80% of insulin exocytosis events via the use of a zinc‐based fluorophore with spinning‐disc confocal microscopy [47]. They identified these β cells as readily releasable β cells (RRβs), which are functionally heterogeneous at the level of exocytosis among β cells. Mechanistically, while glucose concentrations as high as 18.2 mM completely triggered RRβs to release insulin in a synchronized manner during the first phase, even greater glucose concentrations increased the continuous secretion from these cells in the second phase. Similarly, release‐incompetent β cells also presented glucose‐evoked Ca^2+^ transients but exhibited Ca^2+^‐exocytosis coupling deficiency.

#### 2.1.2 In Addition to Glucose, FAs, and Amino Acids Regulate Insulin Release

In addition to GSIS, FAs also stimulate low glucose secretion in pancreatic islet β cells [48]. This study reported that the mitochondrial β‐oxidation of FAs close KATP channels synergically synergizes with elevated ATP and redox signals in the form of H_2_O_2_, which is released from pancreatic islets. On the one hand, H_2_O_2_ promoted nonspecific calcium channel depolarization in the cell and further depolarization of Ca^2^⁺ channels, which all induced Ca^2^⁺ influx and promoted insulin release; on the other hand, H_2_O_2_ activated mitochondrial calcium‐independent phospholipase A_2γ_ (iPLA_2γ_) to supply FAs for G‐protein‐coupled receptor‐40 (GPR40). Although free FAs are considered to augment GSIS by activating GPR40 [49, 50, 51, 52], free FAs also suppress insulin secretion, such as the activation of Toll‐like receptor (TLR)2 and TLR4 in chronic excess [53].

Increased levels of branched‐chain amino acids (BCAAs) in the blood can also trigger insulin release, potentially exhausting the insulin secretory reserve in early T1D [54, 55]. Superoxide formation upon oxidation of BCCAs contributes to insulin secretion and promotes increased β‐cell mass and function by activating the regulatory serine/threonine kinase mammalian target of rapamycin complex 1 (mTORC1) [56]. A variety of substances have the capacity to regulate insulin secretion, either directly or indirectly, through intricate pathways, potentially leading to β‐cellular damage.

### 2.2 Glucagon (α Cells)

**FIGURE 3:**
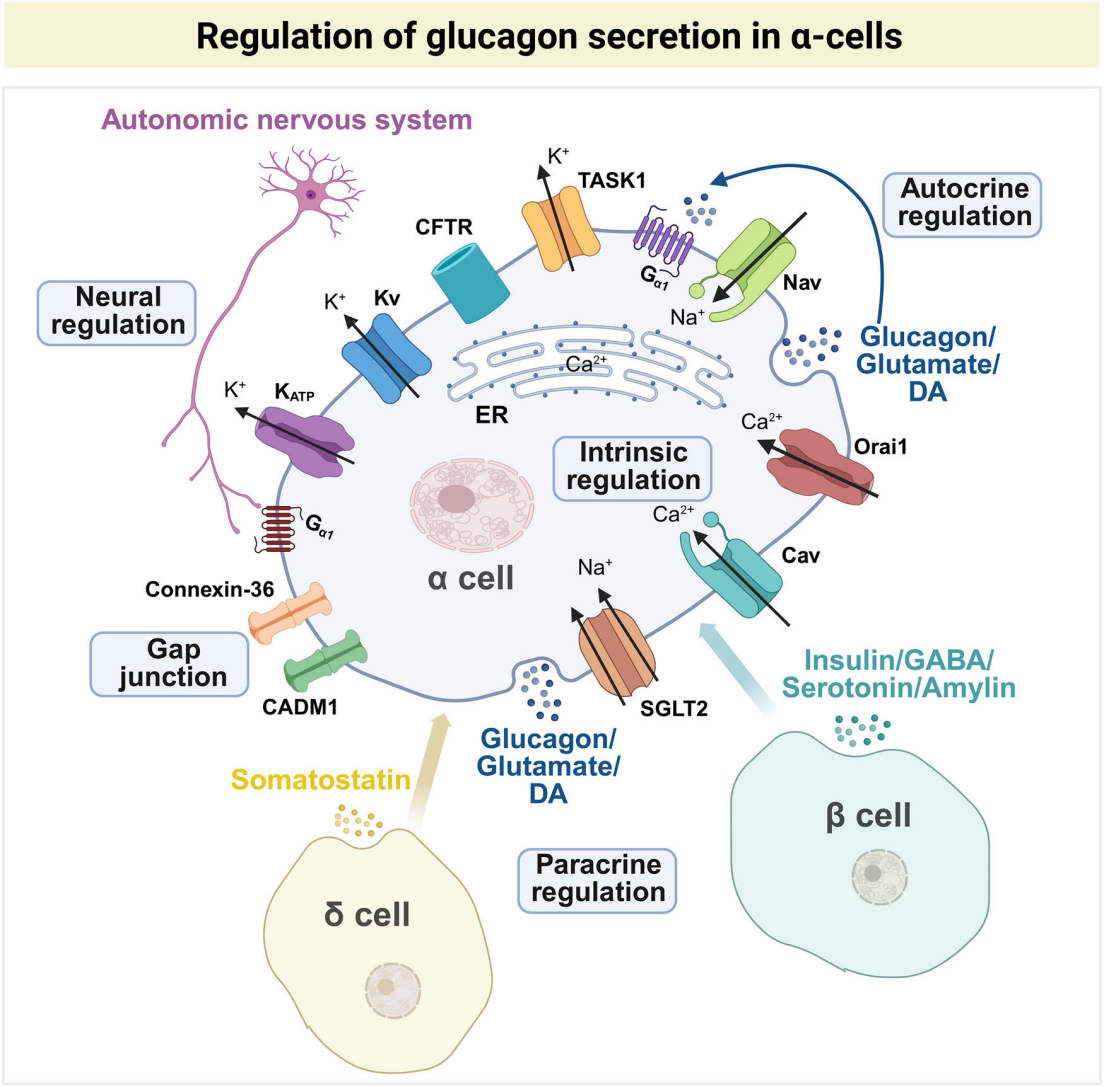
Regulation of glucagon secretion in α cells. α Cells secrete glucagon via several regulatory pathways. Intrinsic regulation: α cells are capable of detecting alterations in intracellular K^+^ concentrations in response to glucose stimulation, and in the absence of glucose, α cells are electrically active and show Ca^2+^ oscillations. Paracrine regulation: Paracrine mechanisms proposed to control glucagon secretion from α cells involve the release of inhibitory factors, including somatostatin from δ cells and insulin/GABA/serotonin/amyin from β cells. Autocrine regulation: Glutamate/DA coreleased with glucagon can potentiate glucagon secretion by increasing the cytoplasmic Ca^2+^ concentration. Neuronal regulation: Sympathetic stimulation enhances glucagon release via the activation of β‐adrenergic receptors on α cells. Direct cell‐to‐cell communication through gap junctions, α cells, and other islet cells achieve direct electrical coupling, which facilitates synchronized responses to stimuli. Created with BioRender.com. DA, dopamine; CFTR, cystic fibrosis transmembrane conductance regulator.

The precise mechanisms underlying glucagon release have yet to be fully elucidated. The proposed mechanisms include intrinsic processes, paracrine and autocrine signaling, neuronal modulation, and direct cell‐to‐cell communication through gap junctions (Figure 3).

#### 2.2.1 Ion Channels

The primary mechanism controlling α‐cell action potential (AP) generation and glucagon secretion involves ion channels, particularly KATP channels [8]. These heteromeric KATP channel complexes are composed of Kir6.2, an inwardly rectifying potassium channel, and SUR1, a sulfonylurea receptor that confers metabolic sensitivity [57, 58]. Unlike β cells, α cell KATP channels exhibit low basal activity due to sustained ATP production, even under conditions of low glucose availability, facilitated by high‐affinity glucose transporters (GLUT1/SGLT1) and the highly efficient glycolytic enzyme hexokinase‐1 [59, 60, 61]. An increase in glucose levels reduces α‐cell KATP conductance, leading to membrane depolarization and the suppression of APs, thereby inhibiting glucagon secretion [59, 62].

In addition to KATP channels, α cells utilize other distinct ion channels to regulate glucagon secretion. α Cells rely on voltage‐gated Nav1.3 and Nav1.7 channels for AP initiation, with their inactivation properties shaping electrical activity [63]. P/Q‐type Ca_v_ channels predominantly mediate exocytosis, whereas L‐type Ca_v_ channels play a key role during adrenergic stimulation [59, 64, 65]. Kv2.1 and BK potassium channels enable rapid repolarization, maintaining AP firing [66, 67]. Additional regulators include TASK1 [68] (a glucose‐activated hyperpolarizing K⁺ channel), Orai1 [69] (store‐operated Ca^2^⁺ entry), and cystic fibrosis transmembrane conductance regulator (CFTR [70]) (a cyclic AMP [cAMP]‐dependent Cl^−^ channel), which dampen excitability, whereas sodium‐glucose cotransporter 2 (SGLT2) [71] (an electrogenic Na⁺/glucose cotransporter) and CAT2 [66] (arginine‐induced depolarization) stimulate secretion. Nutrient sensing further involves glycine‐gated Cl^−^ channels (with context‐dependent effects) and lactate‐induced KATP activation, highlighting the intricate interplay of ion channels in α‐cell stimulus‐secretion coupling [72, 73, 74].

#### 2.2.2 Paracrine and Autocrine Regulation

Paracrine interactions within islets significantly influence glucagon secretion. Insulin, which is secreted by β cells, potently inhibits glucagon release [75]. Insulin binds to insulin receptors on α cells, activating downstream signaling pathways such as phosphoinositide 3‐kinase/Akt, which leads to decreased cAMP levels and reduced glucagon secretion [76]. Somatostatin, produced by δ cells, also inhibits glucagon secretion by decreasing cAMP levels through the inhibition of adenylate cyclase activity [77, 78]. Furthermore, γ‐aminobutyric acid (GABA) [79], serotonin [80], and amylin [81, 82], which are cosecreted with insulin from β cells, contribute to the suppression of glucagon secretion.

In addition to intraislet paracrine signaling, α cells have been shown to regulate glucagon secretion through autocrine mechanisms. Glucagon binds to glucagon receptors to promote α‐cell exocytosis and cAMP production [83]. Additionally, α cells release glutamate, which, by activating glutamate receptors as a positive autocrine signal, enhances glucagon secretion via membrane depolarization, Cav channel activation, and an increase in the cytoplasmic free Ca^2^⁺ concentration [84]. Furthermore, it has been proposed that α cells locally synthesize catecholamines, with postprandially synthesized dopamine (DA) acting through autocrine/paracrine signaling to modulate glucagon secretion [85].

#### 2.2.3 Neural Regulation

The autonomic nervous system exerts significant control over glucagon secretion. Sympathetic stimulation enhances glucagon release via the activation of β‐adrenergic receptors on α cells [86]. The activation of these receptors leads to increased intracellular calcium levels, promoting glucagon exocytosis [65, 86]. Parasympathetic stimulation, which is mediated by vagal nerve activity, generally stimulates glucagon secretion through muscarinic receptors [87]. In addition, nicotine influences α‐cell glucagon secretion through specific ionotropic cholinergic receptor activation, which is part of the parasympathetic nervous system's regulation of pancreatic islet hormones [88].

#### 2.2.4 Direct Cell–Cell Contact via Gap Junctions

Gap junctions between α cells and other islet cells facilitate direct electrical coupling, enabling synchronized responses to stimuli. The adhesion molecule CADM1 facilitates gap junction communication among pancreatic islet α cells and helps regulate glucagon secretion, preventing excessive release [89]. Additionally, β cells and δ cells are connected via gap junctions mediated by connexin‐36, which contributes to the inhibition of glucagon secretion [90].

### 2.3 Other Islet Hormones

Given the limited abundance of δ cells, ε cells, and PP cells, the cellular and molecular mechanisms regulating their hormone secretion remain poorly understood. The current state of knowledge regarding these mechanisms is summarized below.

#### 2.3.1 Somatostatin (δ Cells)

Despite comprising only a small fraction of islet cells, δ cells play a crucial role in maintaining balanced hormonal outputs within pancreatic islets. Their intricate morphology, characterized by extensive projections, facilitates interactions with other endocrine cells, increasing their regulatory impact [9, 91]. These δ cells exhibit electrical excitability, and similar to β cells, K_ATP_ channels are pivotal in translating metabolic signals into changes in the membrane potential [92]. The glucose‐stimulated SST secretion process involves complex Ca^2^⁺ signaling pathways, including both voltage‐gated Ca^2^⁺ entry and nonelectrogenic mechanisms such as endoplasmic reticulum Ca^2^⁺ mobilization and Na⁺‐Ca^2^⁺ exchanger activity [93, 94].

In addition to their intrinsic regulatory mechanisms, δ‐cell SST secretion is finely modulated by neighboring islet cells through paracrine regulation. β‐Cell‐derived factors, including insulin [95, 96], urocortin‐3 [97], and GABA [98,] exert stimulatory effects through SGLT2, CRHR2, and GABA_a_ receptor mechanisms, respectively, whereas serotonin provides inhibitory effects via 5‐HT5A receptor‐mediated cAMP reduction [80]. Electrical coupling through connexin‐36‐containing gap junctions synchronizes δ‐cell activity with that of neighboring β cells [66, 99]. Similarly, α cells contribute to δ‐cell regulation through multiple pathways involving glucagon receptor activation, glutamatergic signaling via AMPA receptors, cholinergic inputs through M1 receptors, and the potential actions of cosecreted glicentin‐related PP [9, 100, 101, 102].

Autocrine regulatory mechanisms further fine‐tune δ‐cell function, with somatostatin itself providing negative feedback through highly expressed somatostatin receptor (SSTR)1 and SSTR3 receptors in δ cells [9, 103, 104]. Additional modulation occurs through neuronostatin signaling via GPR107 and cortistatin, which act through both somatostatin and ghrelin receptors [105, 106, 107, 108].

#### 2.3.2 ε Cells and PP Cells

The pancreatic ε‐cell population, first characterized as a unique endocrine subset in the first decade of the 21st century, produces the metabolically active peptide ghrelin, which requires n‐octanoylation at serine 3 for biological activity [109, 110]. Although the complete regulatory mechanisms governing ghrelin biosynthesis and secretion remain to be fully elucidated, the current understanding reveals several critical transcription factors, including Nkx2.2, Pax4, Pax6, and Arx, that coordinately regulate ε‐cell development and functional maintenance [16, 111, 112, 113, 114]. These specialized endocrine cells secrete both acylated (AG) and unacylated (UAG) forms of ghrelin, with the AG isoform mediating its primary effects through binding to the growth hormone secretagogue receptor GHS‐R1a [115, 116, 117]. Through paracrine signaling mechanisms, ε‐cell‐derived AGs modulate pancreatic islet function via both direct and indirect pathways directly through the inhibition of β‐cell cAMP signaling and potassium channel activation and indirectly via the stimulation of somatostatin release from δ cells, which subsequently suppresses insulin secretion [66, 118, 119]. In contrast, the UAG isoform has distinct physiological activities, including increased insulin sensitivity and protection of β‐cell viability [120, 121, 122, 123]. Further research is needed to fully elucidate the regulatory networks controlling ε‐cell ghrelin secretion and its integration with overall islet function.

PP was initially identified in birds and subsequently isolated from the pancreata of various mammalian species by Chance and Jones in 1974 [124]. PP cells constitute a unique endocrine population that synthesizes a neuropeptide belonging to the NPY family [125]. Postprandial secretion of PP occurs rapidly, primarily through neural regulatory mechanisms, as evidenced by a significant reduction following vagal nerve disruption, with cholinergic neurotransmission playing a central role in this process [126, 127]. Critically, PP directly inhibits islet hormone secretion, suppressing insulin, glucagon, and somatostatin release through paracrine signaling [128, 129, 130]. These findings position PP as a key modulator of islet cross‐talk, with disrupted secretion potentially contributing to diabetic hyperglucagonemia or impaired insulin responses. Furthermore, PP‐rich pancreatic regions display significantly reduced β‐ and α‐cell masses, suggesting structural implications for diabetes progression [131]. PP secretion is additionally modulated by neighboring ε cells via ghrelin's inhibitory action through the GHS‐R receptor, highlighting PP as a potential target for modulating islet function in diabetes [132, 133].

## 3 Functional Crosstalk in Islet Physiology

**FIGURE 4:**
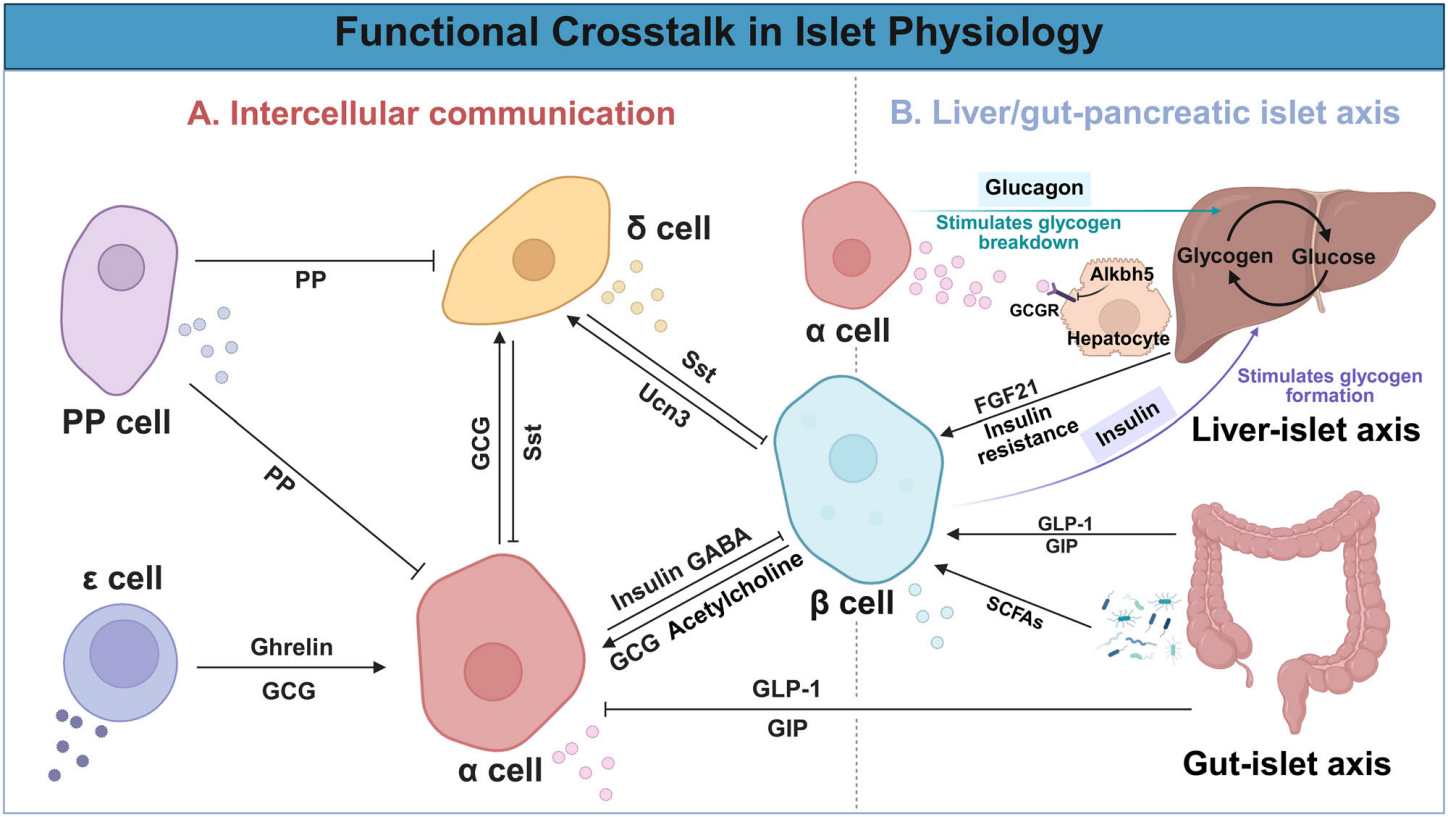
Functional crosstalk in islet physiology. (A) Intraislet communication: Paracrine secretion is an essential regulatory process that enables communication between various secretory cells in the islets. (B) Liver/gut–pancreatic islet axis: The regulation of blood glucose involves a collaborative effort among various tissues within the human body, including the liver and gut. Created with BioRender.com. SCFAs, short‐chain fatty acids; FGF21, fibroblast growth factor 21; Alkbh5, alkylation repair homologue protein 5.

Pancreatic islet function is regulated by glucose and other metabolic signals, with increasing evidence highlighting the roles of intraislet communication and interorgan signaling in maintaining glucose homeostasis. These mechanisms involve paracrine factors, neurotransmitters, and electrical coupling that coordinate with hormone secretion. In addition, peripheral organs such as the liver and gut influence islet activity through endocrine and neuroendocrine pathways (Figure 4).

### 3.1 Intercellular Communication

Paracrine secretion constitutes a crucial regulatory mechanism that facilitates the interrelation among diverse secretory cells within islets. For example, the classical GSIS is the outcome of glucose stimulating β cells, which is enhanced by the amplifying effects of peptide hormones from α cells but limited by the inhibitory effects of somatostatin from δ cells [134].

The function of glucagon in intraislet paracrine regulation is crucial. Within the islet, it is apparent that endogenous glucagon increases insulin secretion under hyperglycemic conditions. The paracrine actions of glucagon are essential for sustaining normal insulin release, and intraislet glucagon signaling includes the activation of both the GCGR and GLP‐1R [135]. Wei et al. reported that GCGR antagonism promotes β‐cell regeneration and that the inhibition of glucagon‐GLP‐1R signaling reduces the insulin‐stimulating effect and β‐cell regeneration caused by a GCGR monoclonal antibody in diabetic mice [136]. Therefore, in addition to GCGR, GLP‐1 can also potentiate GSIS, mainly by activating the β cell Glp1r, whereas Gcgr, which has a lower affinity for GLP‐1 than glucagon does, primarily stimulates increased glucose production in the liver [135, 137].

In contrast, somatostatin, discovered in pancreatic δ cells, is recognized as a potent inhibitor of insulin and glucagon secretion [15]. Somatostatin secretion is stimulated by glucose, and the reaction of δ cells to glucose follows the rhythmic and coordinated response of pancreatic β cells, all of which suggest that β‐ and δ cells coordinate through direct gap junctions [90, 138]. van der Meulen et al. [97] reported another regulatory mechanism in which the peptide hormone urocortin 3 (Ucn3) is costored and released with insulin, increasing glucose‐induced somatostatin secretion through specific receptors on δ cells. This study revealed that the paracrine effects of Ucn3 trigger a negative feedback loop that stimulates somatostatin release, which ensures that insulin secretion decreases once plasma glucose levels normalize. Therefore, the paracrine regulatory mechanism of somatostatin facilitates the timely reduction in peripheral insulin levels once peripheral blood glucose concentrations normalize, thereby preventing the onset of hypoglycemia. Somatostatin also acts in a paracrine manner to suppress α‐cell activity, with antagonists of somatostatin increasing glucagon output and preventing glucose‐induced suppression of secretion [77]. B. K. Lai et al. [139] reported that the glucagonostatic effect of somatostatin is mediated by two types of SSTRs, SSTR2 and SSTR3, and that the ability of glucose to inhibit glucagon is only partially reliant on paracrine inhibition by somatostatin.

GABA is present in pancreatic β cells in addition to the central nervous system, and its effect on the islet cell network is contingent upon its interaction with GABA receptors, which are detected on the rodent and human α‐cell membrane surface [79, 140, 141]. Research conducted as early as 35 years ago demonstrated that GABA, released concurrently with insulin, may play a role in mediating the inhibitory effect of glucose on glucagon secretion, which is achieved through the activation of GABAA‐receptor Cl^−^ channels in α cells [142]. Feng et al. reported that treatment with GABA in STZ‐induced diabetic mice largely restored the immunodetectable levels of insulin and GAD in β cells and significantly decreased the number of aldehyde dehydrogenase 1 family member A3 (ALDH1a3)‐positive cells, the α‐cell mass and hyperglucagonemia [143]. Therefore, the potential for GABA to induce the transdifferentiation of α cells into β cells has been a subject of ongoing academic discourse. Upon sustained GABA exposure, α cells are induced to be converted into β‐like cells, which are functional and can repeatedly reverse chemically induced diabetes in vivo [144]. Artemisinins, small molecules that functionally repress Arx by causing its translocation to the cytoplasm, can target GABAA receptor signaling for the regeneration of the pancreatic β‐cell mass from α cells [145]. However, another study revealed that long‐term administration of artesunate or GABA to mice did not stimulate α‐to‐β‐cell transdifferentiation or insulin secretion [146]. Although GABA signaling aids in the regeneration of β cells, curbs excessive activity in α cells, and encourages the conversion of α cells into β cells [147, 148, 149, 150], the potential role of targeting the GABA signaling pathway in clinical treatment requires further investigation. In addition, PP, which is secreted by PP cells, and ghrelin, which is secreted by ε cells, are implicated in the regulation of intercellular paracrine signaling; however, their precise effects remain incompletely understood.

In addition to paracrine secretion, the coupling of electrical signaling between islet cells is also required for glycemic control, and the specific coupling of β cells is established through gap junctions, which are composed mainly of connexin36 [151]. Connexin36 is expressed in different islet cells but not only in insulin‐producing β cells [152]. Therefore, coupling may occur between β cells and other cells, such as α cells [153]. Evidence from studies suggests that there are no functional gap junctions between α and β cells and that the coupling of connexin36 gap junctions in mouse islets is very heterogeneous [154, 155]. Electrical coupling provided by connexin36 gap junctions, such as the transfer of a depolarizing current, the synchronization of KATP channel‐regulated membrane depolarization, and the coordinated [Ca^2+^]i oscillations across the islet, is important in insulin delivery [156, 157]. For example, St Clair et al. reported that modulating gap junction coupling via connexin36 overexpression or pharmacological activation via modafinil could increase connexin36 gap junction coupling and protect against decreases in coordinated [Ca^2+^] dynamics; importantly, S293, a peptide mimetic of the c‐terminal regulatory site of connexin36, rescued gap junction coupling and [Ca^2+^] dynamics in islets from both db/db mice and a subset of T2D donors [158]. Therefore, targeted gap junction coupling is a potential direction for regulating glucose.

### 3.2 Liver/Gut–Pancreatic Islet Axis

The regulation of blood glucose homeostasis is not exclusively governed by the pancreatic islets; rather, it involves a collaborative effort among various tissues within the human body, including the liver and small intestine [159, 160]. For example, the liver and pancreatic islets contribute twofold to the maintenance of euglycemia. Islet‐associated hormones are crucial in the regulation of hepatic glucose uptake, synthesis, and decomposition [161, 162]. Research has developed a new microfluidic multiorganoid system to reveal the human liver–pancreatic islet axis in normal and disease states, and the combined culture of liver and islet organoids results in beneficial growth and improved functions specific to the tissues [163]. Fibroblast growth factor 21 (FGF21), which is expressed mainly in the liver and whole pancreas, has been shown to regulate glucose homeostasis, such as stimulating glucose uptake into adipocytes and inhibiting hepatic glucose output [164, 165, 166]. One study reported that FGF21, along with GLP‐1, contributes to the prevention of insulin‐induced diabetes in mice without glucagon action. Interestingly, Cui et al. reported that liver‐derived FGF21 was related to GCGR antagonism‐induced β cell regeneration in a T2D mouse model [168]. The use of the FGF21 nAb to block FGF21 activity diminished the upregulation of characteristic genes associated with β‐cell identity in the plasma or hepatocytes of GCGR mAb‐treated mice. Ding et al. reported that hepatocyte‐specific deletion of alkylation repair homologue protein 5 (Alkbh5) reduces glucose and lipid levels by inhibiting the GCGR and mTORC1 signaling pathways and that targeting Alkbh5 could reverse T2DM in diabetic mice [169]. In addition, the sequential relationship between hepatic insulin resistance and β‐cell dysfunction also needs to be further elucidated. Although the initially prevailing view is that insulin resistance causes increased plasma glucose levels, which promote increased demand for pancreatic β cells to produce and secrete more insulin, a growing awareness exists that, at least for a subset of patients, long‐term heavy intake of high‐carbohydrate and high‐fat foods causes islet β‐cell hyperresponsiveness, which contributes to some other disorders associated with metabolic syndrome, including nonalcoholic fatty liver disease [170].

In addition to the liver, the “gut–islet axis” also serves as a vital endocrine signaling pathway, managing islet function via the interaction between the intestinal microecology and endocrine metabolism [171]. Recent research has clearly shown a two‐way communication pathway between the gut and islets, allowing the gut to significantly impact glucose metabolism and energy balance in animals, especially GLP‐1 and glucose‐dependent insulinotropic polypeptide (GIP). Specifically, GLP‐1, which is released by intestinal L cells, has a dual regulatory effect on pancreatic cells by increasing insulin release from β cells in a glucose‐dependent manner and suppressing glucagon release from α cells, highlighting its important regulatory role. In addition, short‐chain FAs (SCFAs), which are produced by the gut microbiota through the fermentation of indigestible carbohydrates, are closely associated with the development of T2D [172]. A study highlighted the potential beneficial effects of SCFAs in preventing T2DM development [173]. They reported that the majority of other potential SCFA‐producing bacterial strains were either diminished or remained unaltered in individuals with T2DM. Conversely, when SCFA‐producing bacteria, promoted by dietary fiber, exhibited increased diversity and abundance, there was a more significant improvement in the participants’ hemoglobin A1c levels. On the basis of the biological mechanism of SCFAs, the absence of GPR43, an SCFA receptor, and its expression on pancreatic β cells might result in reduced glucose tolerance and a decrease in pancreatic β‐cell mass in mice [174]. In addition to metabolic pathways, microbial components, such as LPS, influence islet function via inflammatory processes through being sensed by specific pattern recognition receptors, including TLRs. Some studies have indicated that TLR signals mediate islet function although islet macrophages or cell cycle regulators [175, 176]. However, the intricate features of the gut necessitate further comprehensive investigation to elucidate additional mechanisms of the “gut–islet axis” in the regulation of glucose levels in the future, especially the gut microbiota.

## 4 Islet Hormone Dysfunction in Diabetes

While it is commonly accepted that the primary cause of diabetes is insufficient insulin secretion or impaired insulin function, emerging evidence suggests that dysfunction of various hormones within the pancreatic islets collectively contributes to the onset and progression of diabetes through multiple mechanisms [177]. This section discusses the changes in islet hormone secretion and function that occur during the development of T1D and T2D.

### 4.1 Type 1 Diabetes

T1D is an autoimmune disorder caused by the destruction of insulin‐producing β cells in the pancreatic islets, typically resulting in a significant lack of endogenous insulin, and lifelong insulin therapy is needed for survival [178]. Numerous studies have elucidated the specific mechanisms underlying autoimmune damage to β cells. For example, one hypothesis posits that viral infection induces an inflammatory response within islets, but endogenous double‐stranded RNA in β cells could lead to a diabetogenic immune response, thus identifying a virus‐independent mechanism for T1D initiation [179]. Mechanistically, the disruption of ADAR, an RNA editing enzyme in β cells, initiates a large interferon response, islet inflammation, and β‐cell failure and destruction, similar to early‐stage human T1D. Theoretically, the inadequate secretion of insulin resulting from damage to β cells can be managed through continuous insulin supplementation in individuals with T1D, but relying solely on insulin as a treatment does not fully resolve all problems, which suggests that the dysregulation of additional hormones significantly contributes to the progression of T1D [180, 181]. Noninsulin adjuvant therapy, especially GLP‐1, has significant potential in clinical applications [182]. For example, Jin et al. reported that excessive intestinal GLP‐1 is strongly associated with impaired counterregulatory responses to hypoglycemia in patients with T1D [183]. Enhancing the functionality of other hormones represents a significant approach for adjunctive therapy in managing complications associated with T1D.

### 4.2 Type 2 Diabetes

Dysfunction of islet hormones constitutes a significant pathogenic factor in the development of T2D, which is characterized by hyperglycemia, insulin resistance, defective insulin secretion, and loss of β‐cell function and mass. From a genomic perspective, islet epigenomic and transcriptomic analyses are progressively defining the regulatory potential of potential target genes whose dysfunction is likely to contribute to T2D [184, 185, 186, 187, 188]. For example, Gaulton et al. [186] identified 49 distinct association signals at these loci, including five mapped signals in or near KCNQ1, through fine mapping of 39 established T2D loci in 27,206 cases and 57,574 controls of European ancestry [187]. Importantly, the variants most likely responsible for each distinct signal were located primarily in noncoding sequences, suggesting that their association with T2D occurs through gene regulation. Other studies have suggested that regulatory noncoding RNAs may contribute to diabetes progression and β‐cell dysfunction. Fadista et al. [188] reported that 17 long noncoding RNAs (lncRNAs) are abnormally expressed in relation to glycated hemoglobin (HbA1c) levels. Expression quantitative trait loci (eQTLs) were identified for two of these lncRNAs, LOC283177 and SNHG5, but the eQTL single nucleotide variants did not coincide with those for T2D [189]. Using the Illumina 450BeadChip, DNA methylation profiling of 15 T2D and 34 ND islets revealed 1649 CpG sites with differential methylation across 853 genes, with 17 located in T2D‐associated regions [190]. These studies indicate that genomic variation plays a significant role in islet dysfunction and the pathogenesis of T2D; however, the precise mechanisms underlying these associations require further investigation.

From the perspective of the cellular and functional heterogeneity of pancreatic islets, gaining a deeper understanding of the subsets of β cells and their respective functions represents a crucial avenue for investigating the pathogenesis of islet hormone dysfunction and T2D. These genes constitute four subgroups with varying levels of ST8SIA1 and CD9; five groups identified by the expression of RBP4, FFAR4/GPR120, ID1, ID2, and ID3; and groups marked by ER stress‐related and oxidative stress‐related genes [191, 192, 193, 194]. In addition to β cells, recent research highlighting the roles of α‐ and δ cells in influencing β‐cell function and resilience, as well as their involvement in T2D pathogenesis, has sparked renewed interest in these cell types [195, 196]. For example, when stimulated by kainate or a decrease in glucose levels, α cells in humans express the vesicular acetylcholine transporter and release acetylcholine, which enhances the β‐cell response to increased glucose [195]. Therefore, comprehending the molecular repertoire of each islet cell type and prioritizing the classification and enrichment of these cell type populations for transcriptomic analysis are essential.

Other factors, such as islet inflammation, contribute to islet dysfunction. Islet inflammation is characterized by cytokines, immune cells, β‐apoptosis, amyloid accumulation and fibrosis, the typical pathological features of islet dysfunction, which are associated with decreased β‐cell function and impaired insulin secretion [194]. Ehses et al. reported that immune cell, cytokine, and chemokine levels increase in individuals with islet dysfunction, and animal studies revealed that immune cells infiltrate the islets [197]. The role of local tissue inflammation in the pathology of insulin resistance and β‐cell failure in patients with islet dysfunction is gaining increased recognition [198]. Therefore, understanding the role of islet inflammation in the dysfunction of pancreatic hormones, which contributes to the development of T2D, necessitates an examination from the perspective of immune cells and associated factors, including islet amyloids. IL‐1b, TNFa, and IFNc are three key cytokines that likely collaborate during pancreatic immune infiltration to cause β‐cell damage and apoptosis, significantly contributing to diabetes pathogenesis [199, 200, 201]. Islet amyloid decreases glucose uptake mediated by insulin and hinders insulin release in β cells. Gurlo et al. reported that islet amyloid can trigger inflammatory cells to release inflammatory substances such as interleukin (IL)‐1a, potentially causing β‐cell death [202]. Similarly, islet amyloid polypeptide (IAPP), a protein responsible for amyloid deposition in the pancreas in T2D, activates the NLRP3 inflammasome and produces mature IL‐1β [203]. Glyburide, a treatment for T2D, inhibited the production of IL‐1β mediated by IAPP in vitro. Cystic fibrosis‐related diabetes is also an extrapulmonary complication of cystic fibrosis. It occurs due to abnormal glucose metabolism, primarily characterized by insulin deficiency and occasional resistance to insulin [204, 205]. In summary, a comprehensive understanding of the inflammation‐related factors involved in the pathogenesis and progression of T2D is essential for the development of targeted therapeutic interventions aimed at mitigating its progression.

## 5 Therapeutic Strategies Targeting Islet Hormones

The development of therapeutic interventions for diabetes mellitus has undergone a remarkable evolution, progressing from crude animal‐derived insulin extracts to sophisticated molecular therapies that precisely target islet hormone physiology. This section comprehensively examines contemporary treatment strategies, incorporating recent breakthroughs and emerging technologies that promise to transform diabetes care.

### 5.1 Insulin‐Centric Therapies

**FIGURE 5:**
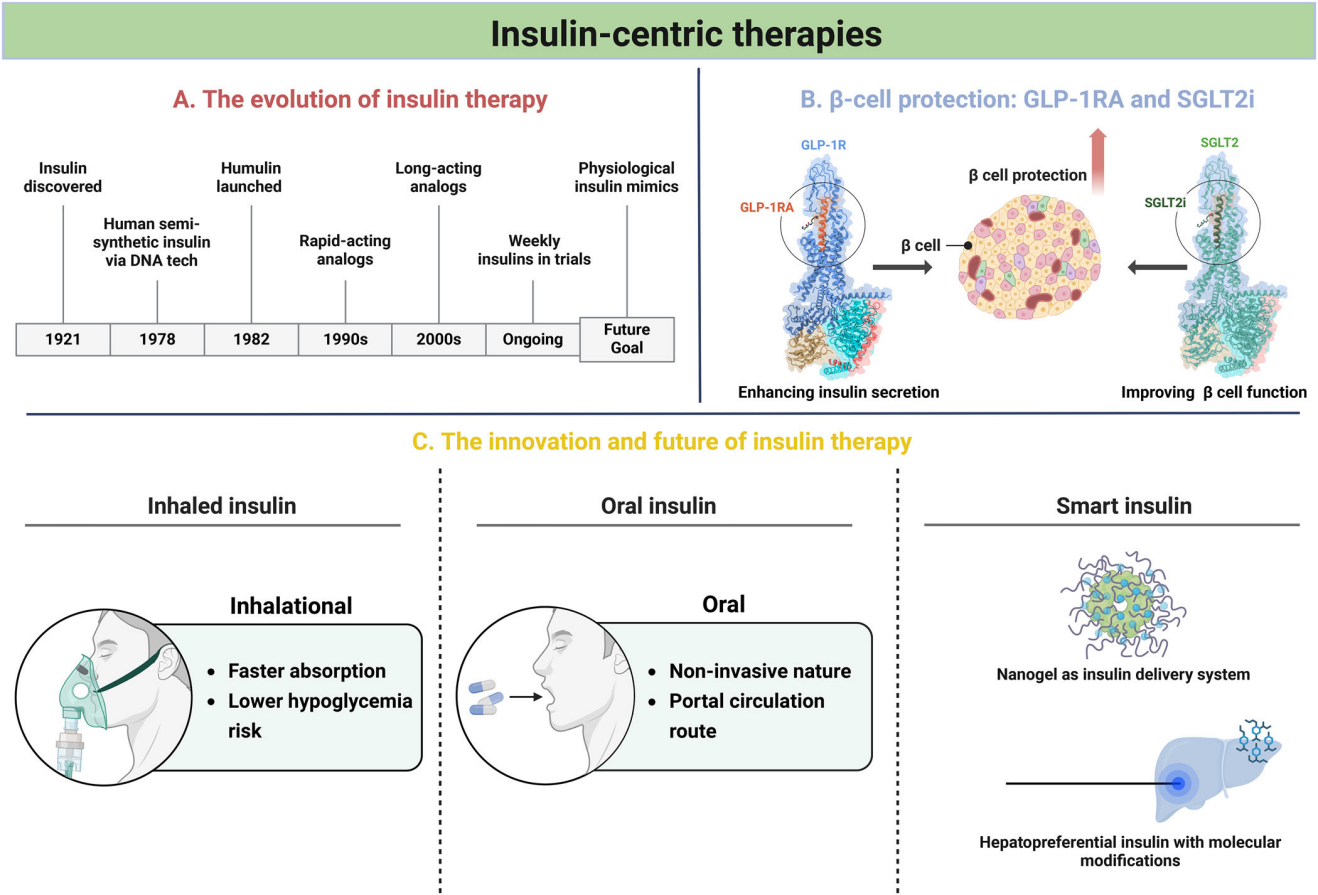
Insulin‐centric therapies. (A) The evolution of insulin therapy. (B) β‐Cell protection with GLP‐1RA and SGLT2i. (C) The innovation and future of insulin therapy include inhaled insulin, oral insulin and smart insulin. Created with BioRender.com. SGLT2i, sodium‐glucose cotransporter 2 inhibitors.

The section on insulin‐centric therapies focuses on three key areas: the evolution of insulin therapy, innovations shaping the future of insulin‐based treatments, and strategies for β‐cell protection (Figure 5).

#### 5.1.1 The Evolution of Insulin Therapy

Insulin therapy has undergone a transformative journey since its discovery in 1921 by Banting and Best [206]. Initially, insulin was extracted from animal sources, primarily bovine and porcine pancreases, leading to issues with immunogenicity and variability in efficacy [207]. The advent of recombinant DNA technology in 1978 enabled the production of human insulin, significantly reducing adverse reactions and improving therapeutic outcomes [206]. In 1982, Eli Lilly brought the first recombinant human insulin, marketed as humulin, to the public [206]. Following this, additional pharmaceutical companies began producing their own human insulin analogues. However, the pharmacokinetics of traditional human insulin require strict adherence to injection schedules and dietary restrictions, posing challenges for patients.

Modern insulin analogues have been developed to address these limitations. Rapid‐acting insulin analogues, such as lispro [208, 209], aspart [210], and glulisine [211], provide a quicker onset and shorter duration of action, allowing for more flexible meal timing. Long‐acting insulin analogues, including glargine [212], detemir [213], and degludec [214], offer stable basal insulin levels with fewer peaks and troughs, thereby enhancing glycemic control and reducing hypoglycemic episodes. Furthermore, weekly insulin analogues such as Icodec [215] and insulin Fc [216] are currently under development, featuring an extended glucose‐lowering effect that is being evaluated in ongoing clinical trials. Despite these advances, the ultimate goal remains the development of insulin formulations that more closely mimic physiological insulin secretion, effectively controlling fasting and postprandial blood glucose levels while minimizing the risk of hypoglycemia.

#### 5.1.2 Innovation and Future of Insulin Therapy

Modern research explores multiple innovative approaches to insulin administration beyond traditional injections. Pulmonary delivery systems represent promising alternatives, with Afrezza being the only United States Food and Drug Administration‐approved inhaled insulin currently available [217]. Compared with subcutaneous injections, this ultrarapid‐acting formulation results in faster absorption and a lower risk of hypoglycemia, although concerns about pulmonary effects persist [218, 219]. Oral administration remains highly desirable because of its noninvasive nature and physiological portal circulation route [206]. Significant advancements in this area include several promising developments: the insulin formulation ORMD‐0801, oral polymersomes designed to promote absorption, and the ingestible self‐orienting millimeter‐scale applicator loaded with insulin [220, 221, 222]. These innovations represent promising strategies for advancing oral insulin delivery, although bioavailability challenges and production costs remain significant barriers.

In addition, researchers are actively developing glucose‐responsive “smart” insulins designed to activate only when blood glucose exceeds physiological thresholds, potentially revolutionizing diabetes treatment through the replacement of more physiological insulin. Among current approaches, phenylboronic acid‐based formulations represent a leading strategy due to their reversible glucose‐binding capacity; these compounds activate insulin release upon hyperglycemia and automatically deactivate when glucose normalizes [223]. This enables diverse applications: Shiino's synthetic polymer gels competitively release insulin when glucose displaces bound insulin [224]; glucose‐sensitive micelles respond rapidly to postprandial spikes; and injectable hydrogels maintain responsiveness for extended periods. Recent advances have further optimized these systems through strategic molecular modifications, such as fluorinated phenyl rings, which increase the stability while preserving the crucial glucose‐responsive properties essential for clinical application [225, 226]. Another innovative direction involves the use of hepatopreferential insulins such as peglispro, which better replicate natural insulin distribution patterns through enhanced hepatic uptake. While these technological advances show tremendous therapeutic potential, critical challenges in clinical translatability remain. Key factors demanding resolution include ensuring reliable bioavailability at the target site, maintaining long‐term stability of the glucose‐sensing moiety and insulin conjugate under physiological conditions, guaranteeing biocompatibility to avoid inflammatory responses or toxicity, and achieving consistent safety and efficacy profiles across diverse patient populations. Skewed insulin dosing remains possible due to various factors influencing blood glucose, and insulin dosing is unlikely to completely eliminate hypoglycemia [227]. Parallel innovative directions, such as the use of hepatopreferential insulins, face similar translational barriers due to adverse hepatic effects, with current research focusing on optimizing liver‐targeted oral formulations and molecular modifications for improved hepatic specificity and safety [206].

However, current insulin replacement therapies face several persistent challenges during prolonged use. The most critical limitation remains the risk of iatrogenic hypoglycemia, particularly with intensive glycemic control, as exogenous insulin administration cannot perfectly replicate physiological glucose‐responsive secretion. Additionally, chronic insulin therapy may contribute to β‐cell exhaustion through a paradoxical feedback mechanism—persistent peripheral hyperinsulinemia can downregulate endogenous insulin production while accelerating β‐apoptosis. Weight gain associated with insulin therapy further complicates long‐term management by exacerbating insulin resistance, creating a vicious cycle of escalating dosage requirements.

#### 5.1.3 β‐Cell Protection: GLP‐1RA and SGLT2i

In addition to insulin replacement, protecting and preserving β‐cell function is crucial for long‐term diabetes management.

GLP‐1 receptor agonists (GLP‐1RAs) have demonstrated significant potential in this regard since the introduction of exenatide as a first‐in‐class agent two decades ago [228]. The treatment paradigm has since expanded to include various administration options, ranging from daily to weekly formulations in both injectable and oral preparations [229, 230]. Particularly noteworthy is tirzepatide, a novel dual GLP‐1/GIP receptor agonist that has shown superior efficacy in glycemic control and weight reduction [231, 232, 233]. Mechanistically, GLP‐1RAs improve metabolic outcomes through multiple pathways: enhancing glucose‐dependent insulin secretion, suppressing inappropriate glucagon release, and promoting satiety [234, 235, 236]. Clinical evidence supports their therapeutic value, with a randomized trial demonstrating that more than 50% of patients with suboptimal glycemic control on insulin lispro achieved insulin independence after transitioning to weekly albiglutide therapy, leading to improved outcomes in diabetes management [237]. Nevertheless, safety considerations for clinical deployment include gastrointestinal effects, pancreatitis risk, and agent‐specific thyroid disorders [238]. Moreover, critical issues such as interpatient heterogeneity and tachyphylaxis require consideration: adherence challenges vary between short‐ and long‐acting GLP‐1RA, whereas long‐acting agents exhibit diminished gastric emptying effects over time owing to receptor desensitization, in contrast with the maintenance effects of short‐acting formulations [230, 239].

SGLT inhibitors (SGLT2is) represent another class of drugs that have been shown to have β‐cell protective effects. Clinical evidence from pooled analyses of three randomized trials evaluating canagliflozin showed consistent improvements in quantitative β‐cell function parameters across different treatment regimens (monotherapy or combination therapy versus placebo/sitagliptin) over 6–12 months [240]. These findings are further supported by studies with dapagliflozin, which similarly demonstrated enhanced β‐cell function and insulin secretion capacity in patients with T2D [241, 242]. However, safety profiles include assessments of renal function, dose‐dependent risks of genital mycotic infections, volume depletion‐related hypotension, and euglycemic diabetic ketoacidosis (DKA) [243]. The incidence of DKA remains low but is clinically significant because of delayed diagnosis caused by atypical normoglycemic presentations [244].

### 5.2 Glucagon Modulation

#### 5.2.1 Glucagon Analogues

Hypoglycemia remains a frequent complication in diabetes management, necessitating the availability of effective countermeasures. Currently marketed glucagon formulations serve as critical interventions for severe hypoglycemic episodes, with their first clinical applications dating back to the 1950s [245]. Traditional injectable preparations, which are administered via subcutaneous or intramuscular routes, require reconstitution procedures owing to the inherent instability of glucagon in aqueous solutions. A significant advancement occurred in 2019 with the authorization of the first nasally administered glucagon formulation [246, 247].

#### 5.2.2 Dual/Triple Agonists: GLP‐1/GCG/GIP Receptor Agonists

Dual or triple agonists targeting gut hormones—including GLP‐1, glucagon (GCG), and GIP—offer a promising therapeutic strategy for diabetes by modulating glucagon signaling. These agents leverage glucagon's ability to suppress appetite and enhance energy expenditure while mitigating its hyperglycemic risks through balanced receptor activation [248]. For example, tirzepatide, a dual GIP/GLP‐1 receptor agonist, has demonstrated superior efficacy in glycemic control and weight loss [249]. Additionally, the integration of glucagon and GLP‐1 receptor signaling has improved diabetes management by promoting weight reduction without causing hyperglycemia [250]. Similarly, the unimolecular triple agonist SAR441255 (GLP‐1/GCG/GIP) outperforms dual agonists in preclinical studies, promoting weight loss and glucose regulation without exacerbating hyperglycemia [251]. However, additional long‐term randomized controlled trials are warranted to assess the sustained efficacy and safety profiles of these dual/triple agonists, particularly across diverse patient populations.

#### 5.2.3 α‐Cell Targeting: GCGR Antagonism and Small Molecule Inhibitors

Targeting α cells directly through GCGR antagonism represents another innovative strategy currently under investigation. GCGR antagonism and small‐molecule inhibitors aim to reduce hepatic glucose output by blocking glucagon action [252]. Preclinical studies have demonstrated promising results, showing significant improvements in glucose homeostasis [253, 254]. Several small molecules, including monoclonal antibodies and antisense oligonucleotides designed to inhibit GCGR gene expression, are currently being explored. Additionally, further details on the use of GCGR antagonists in phase I‐II clinical trials are available [255]. For example, a phase 2 clinical trial evaluated the safety and efficacy of volagidemab, an antagonistic monoclonal GCGR antagonist, which demonstrated a notable reduction in HbA1c and exhibited a tolerable safety profile [256]. Importantly, studies involving several glucagon receptor antagonists—volagidemab [256], RVT‐1502 [157], PF‐06291874 [258], and LY240902 [259]—have shown that the frequency of hypoglycemia is not increased compared with that associated with placebo. Although currently in the trial phase, these therapeutic approaches exhibit substantial promise for enabling more precise control over glucagon activity within diabetes management strategies.

### 5.3 Emerging technologies

**FIGURE 6:**
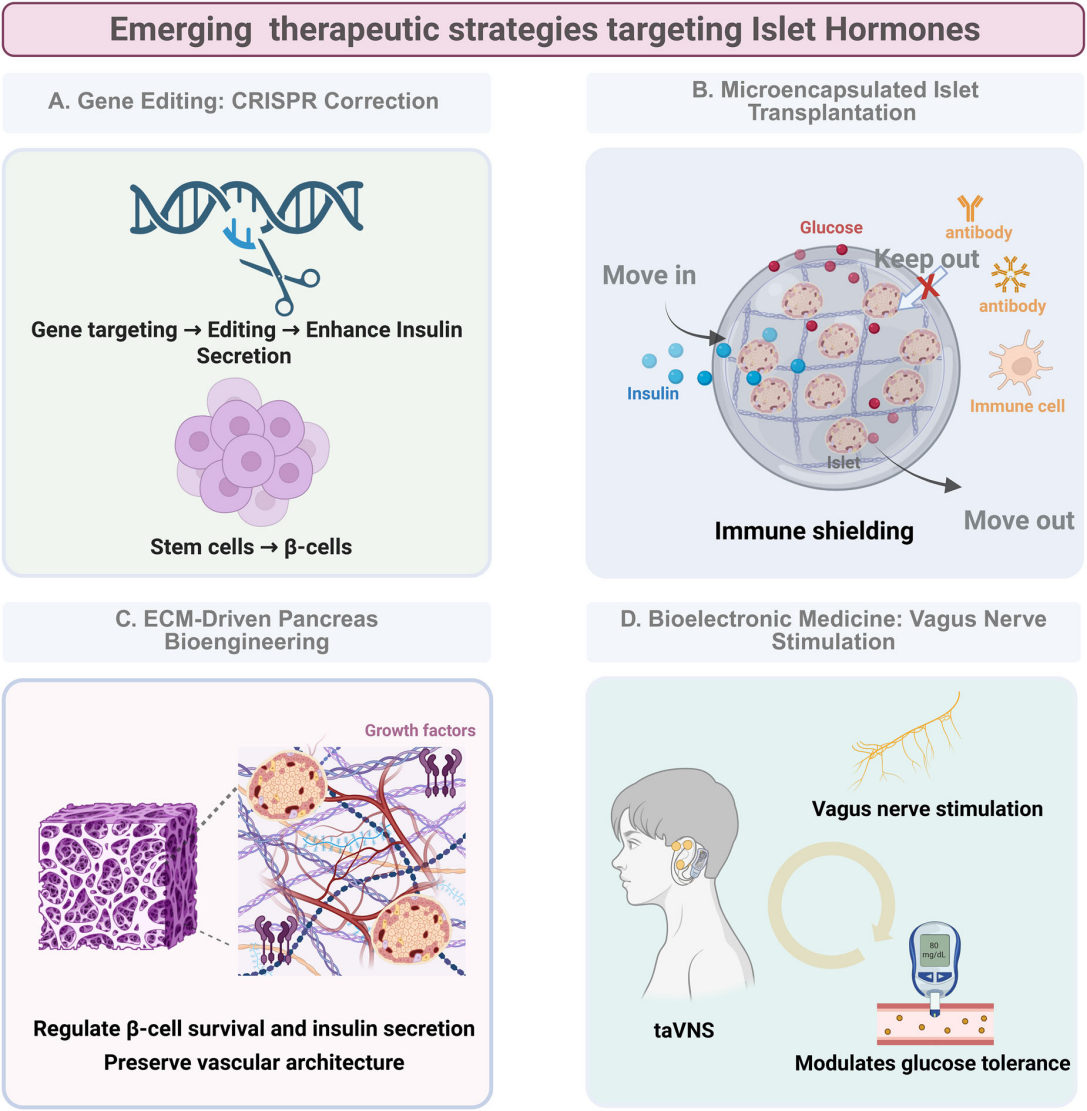
Emerging therapeutic strategies targeting islet hormones. (A) Gene editing with CRISPR correction. (B) Microencapsulated islet transplantation. (C) ECM‐driven pancreas bioengineering. (D) Bioelectronic medicine with vagus nerve stimulation. Created with BioRender.com. ECM, extracellular matrix.

Emerging therapeutic strategies targeting islet hormones include gene editing, microencapsulated islet transplantation, extracellular matrix (ECM)‐driven pancreas bioengineering, and bioelectronic medicine (Figure 6). A comparative analysis of these emerging diabetes therapies is summarized in Table 1.

**TABLE 1:** Comparative analysis of emerging diabetes therapies targeting islet hormones.

Therapy category	Key mechanisms	Efficacy evidence	Delivery/technical challenges	Current clinical stage
Gene editing	Reprogramming of human‐induced pluripotent stem cells CRISPR‐mediated gene editing for insulin sensitivity and insulin secretion CRISPR‐mediated immune protection enhancement	hiPSCs differentiating into functional β cells DPP‐4 knockout restored insulin sensitivity PDX1 editing to promote insulin secretion VCTX211 enhance immune protection and β‐cell survival	Off‐target effect Anti‐Cas9 immune response Inefficient in vivo delivery	Preclinical: murine models (Ref. [261]) Cell lines (Ref. [263]) Phase I/II trials: VCTX210A (NCT05210530) VCTX211 (NCT05565248)
Microencapsulated islet transplantation	Alginate‐based immune shielding for xenogeneic/allogeneic islets Cotransplantation with mesenchymal stem cells to enhance vascularization	Without systemic immunosuppression With sustained insulin independence With improved glycemic control	Fibrosis Site limitations Biocompatible and mechanically stable	Preclinical: Canidae models (Ref. [273]) Murine models (Ref. [272]) Monkey models (Ref. [272]) Porcine models (Ref. [272]) Phase I/II trials: PEG‐encapsulated islets (NCT00260234) Alginate‐encapsulated islets (NCT01739829) Encapsulated human islets (NCT00790257) VC‐01 Combination Product (NCT02239354)
ECM‐driven pancreas bioengineering	ECM preserves native vascular architecture and β‐cell survival cues Synthetic matrices mimic ECM topography to regulate insulin kinetics	With enhanced insulin secretion With stimulated β‐cell regeneration	Biochemical complexity Limited production of organ‐specific decellularized matrices	Preclinical: Cell lines (Ref. [282]) Rat models (Refs. [283, 286]) Canidae models (Ref. [285]) Murine models (Refs. [287, 288])
Bioelectronic medicine	Activation through M3 muscarinic acetylcholine receptors on β cells Noninvasive stimulation restoring autonomic balance	With reduced HbA1c With improved glucose tolerance	Variable patient responses Lack of protocol standardization device interoperability gaps	Preclinical: Rat models (Refs. [293, 294, 296]) Phase I/II trials: taVNS (ChiCTR‐TRC‐12002522)

#### 5.3.1 Gene Editing: CRISPR Correction of Diabetes

Advances in gene editing technologies, particularly CRISPR/Cas9, are reshaping therapeutic strategies for diabetes by addressing their fundamental mechanisms. Central to diabetes pathology is the destruction or dysfunction of insulin‐producing pancreatic β cells, prompting exploration into regenerative solutions such as reprogramming human‐induced pluripotent stem cells (hiPSCs), which are self‐renewing resources capable of differentiating into functional β cells [260]. Parallel advancements target metabolic regulation, exemplified by CRISPR‐mediated silencing of the dipeptidyl peptidase‐4 (DPP‐4) gene via lipid nanoparticle‐delivered Cas9‐sgRNA complexes in diabetic murine models. This approach amplifies endogenous GLP‐1 activity to restore glycemic control and insulin sensitivity [261]. In addition to gene silencing, CRISPR enables precise editing of insulin‐regulating transcription factors, such as pancreatic duodenal homeobox‐1 (PDX1), and coordinates multigene networks to increase β‐cell maturation—simultaneously amplifying glucose‐triggered insulin secretion and prolonging its duration [262, 263]. Besides, the progressive refinement of CRISPR‐engineered β‐cell therapies is exemplified by VCTX211, an allogeneic pancreatic endoderm cell product currently in phase I/II trials (NCT05565248) [264]. This therapy combines CRISPR‐edited stem cells (with B2M and TXNIP knockouts plus PD‐L1, HLA‐E, TNFAIP3, and MANF insertions) with an implantable device to enhance immune protection and β‐cell survival. Furthermore, gene knockouts for generating hypoimmunogenic iPSCs are predominantly achieved using the CRISPR/Cas9 genome editing platform, which employs nucleases such as SpCas9, SpCas9 nickase (SpCas9n), and high‐fidelity variants like HiFi SpCas9 [265, 266, 267]. However, clinical translation faces in vivo safety and efficacy hurdles. Safety concerns include off‐target editing disrupting nontarget genes, on‐target DNA double‐strand breaks causing large‐scale deleterious deletions/rearrangements, and anti‐Cas9 immunity compromising therapeutic safety [260, 268, 269, 270]. Efficient in vivo delivery also remains problematic, necessitating the use of protective vectors (e.g., lipid/chitosan nanoparticles) for targeted cellular entry. Despite these barriers, the curative potential of CRISPR drives ongoing refinement of delivery systems and editing precision.

#### 5.3.2 Microencapsulated Islet Transplantation: Immune Shielding

Islet encapsulation technology offers a promising strategy for the immunoprotection of transplanted insulin‐producing cells while eliminating the need for systemic immunosuppression [271]. This approach involves encasing pancreatic islets within semipermeable biomaterial barriers, enabling nutrient exchange while shielding them from host immune responses. Microencapsulation, which isolates individual islets or small clusters within spherical polymer matrices such as alginate, has demonstrated feasibility in both allogeneic and xenogeneic clinical studies [272]; early results have been promising, with some patients achieving sustained insulin independence and improved glycemic control [273, 274, 275]. Material selection critically influences encapsulation efficacy, with high‐mannuronic alginate emerging as optimal owing to its biocompatibility, selective permeability, and oxygen diffusion capacity [276]. Implantation sites also impact outcomes; for example, subcutaneous and renal capsule placements demonstrate superior islet survival compared with the immunologically active peritoneal cavity [277]. Despite advancements, clinical translation requires resolving fibrotic responses to capsules and standardizing alginate purity to minimize foreign body reactions [278, 279]. Current research prioritizes combinatorial strategies, including cotransplantation with mesenchymal stem cells to increase vascularization, positioning encapsulation as a versatile platform for future β‐cell replacement therapies [280].

#### 5.3.3 ECM‐Driven Pancreas Bioengineering: Regulating Islet Hormones

The ECM of the pancreas, which has far surpassed its historical perception as a passive scaffold, orchestrates critical endocrine functions through dynamic biochemical and biomechanical interactions with islet cells. This three‐dimensional network regulates β‐cell survival, glucose‐responsive insulin secretion, and tissue architecture via structural proteins, sequestered growth factors, and mechanotransduction signaling [281, 282, 283]. Mechanistic studies have revealed that ECM components such as collagens and glycosaminoglycans prevent β‐cell anoikis while enhancing basal insulin release, with topographical organization directly modulating secretory kinetics [284, 285, 286]. Furthermore, ECM‐bound growth factors such as TGF‐β1 govern islet development and regenerative pathways [287, 288]. Emerging bioengineering strategies aim to recapitulate these ECM properties via the use of synthetic or decellularized matrices. While synthetic carriers (e.g., PLGA and PEG) offer tunable mechanical properties, organ‐specific decellularized ECMs better preserve the native vascular architecture and biochemical cues critical for islet cell function [289].

#### 5.3.4 Bioelectronic Medicine: Vagus Nerve Stimulation for Pancreatic Function Regulation

Bioelectronic medicine, specifically vagus nerve stimulation (VNS), offers a novel approach to modulating islet hormone secretion. The principle of VNS for glucose control involves parasympathetic activation through M3 muscarinic acetylcholine receptors on β cells, triggering insulin release while suppressing hepatic glucose production [290, 291]. Targeted pancreatic VNS modulates this pathway by selectively activating vagal branches that innervate the pancreas, while transcutaneous auricular VNS (taVNS) achieves similar effects by stimulating auricular vagal fibers connected to the nucleus tractus solitarius. By electrically stimulating the vagus nerve, VNS provides a nonpharmacological intervention for diabetes management. Preclinical studies in streptozotocin‐induced diabetic rodent models revealed that targeted VNS enhances dynamic blood glucose responsiveness [292]. Research has shown that vagus nerve electrostimulation effectively modulates glucose tolerance in hyperglycemic rats [293]. Translational applications have emerged through taVNS, the noninvasive modality that targets the ear's vagal innervation zones [294]. Clinical evidence indicates that taVNS reduces glucose intolerance and regulates insulin secretion [295, 296]. While these findings highlight neuromodulation's potential as a metabolic intervention, conclusive validation of its antidiabetic efficacy requires more direct evidence and large‐scale trials. Current limitations of VNS for glucose control include species‐specific differences in islet architecture and innervation, which suggest that neuromodulation parameters effective in rodents may not translate directly to humans [292]. Additional limitations include variable patient responses in clinical trials (with only some patients showing parasympathetic enhancement), a lack of standardized stimulation protocols across studies, and device interoperability gaps.

## 6 Challenges and Future Perspectives

**FIGURE 7:**
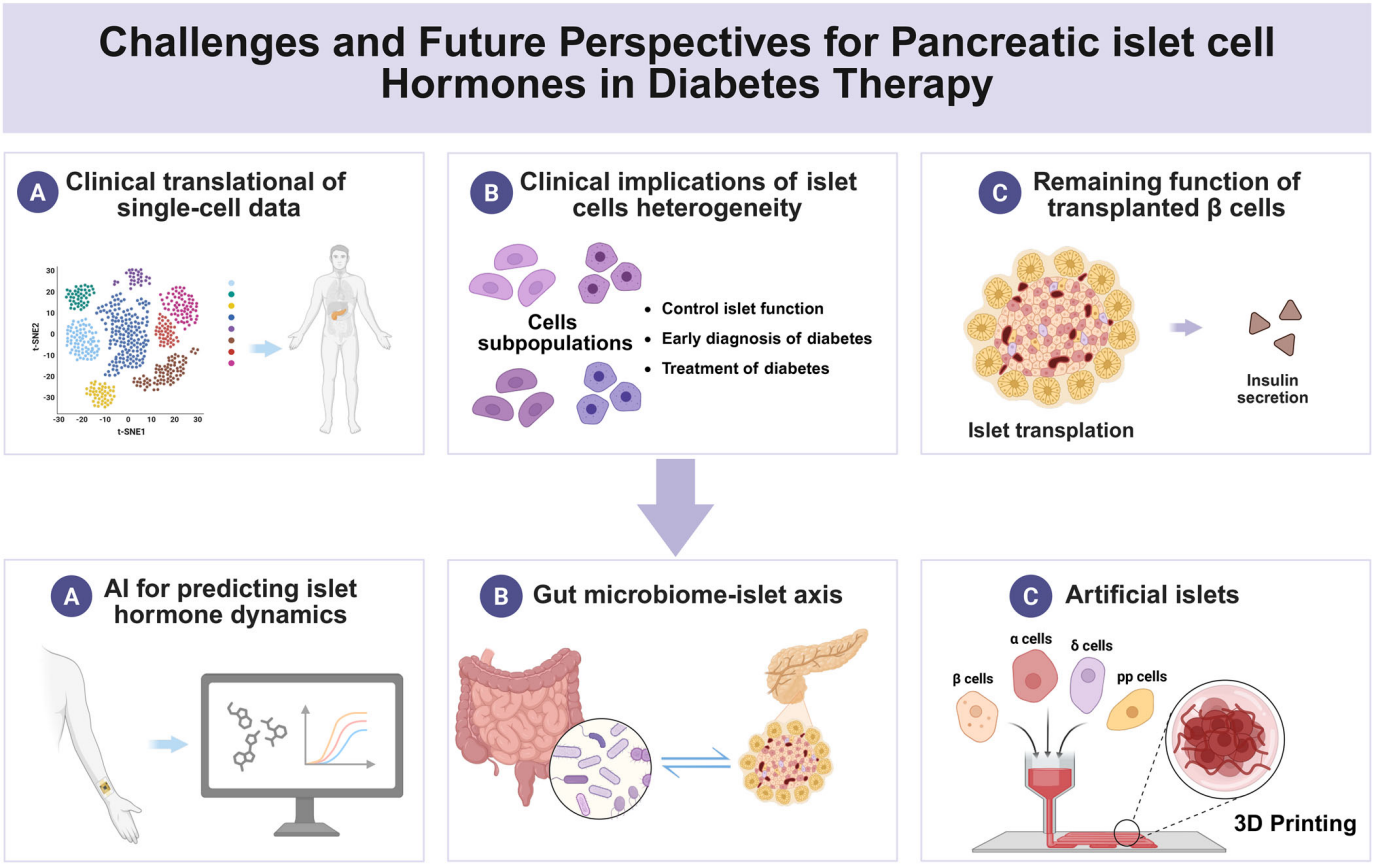
Challenges and future perspectives for pancreatic islet cell hormones in diabetes therapy. Challenges: (A) Single‐cell data for the clinical management of diabetes; (B) implications of islet cell heterogeneity for clinical practice; (C) strategies for preserving cellular function following islet transplantation. Future perspectives: (A) The dynamic secretion of islet hormones was observed through the application of artificial intelligence; (B) clarification of the direct mechanism linking the gut microbiota and pancreatic islets; (C) artificial islets, especially 3D printing technology, for islet transplantation. Created with Biorender.com.

Recent advancements in techniques and methodologies are elucidating the intrinsic regulatory mechanisms of the islet, encompassing the interrelationships among islet cells, the regulation of secretion, and cellular heterogeneity and plasticity. Expanding our understanding of secretion and function of islet cell hormones offers new strategies for avoiding or reversing the dysfunction or loss of islets and the negative effects of hormonal imbalance in diabetes. Nevertheless, numerous challenges remain that require further investigation to fully realize its potential for clinical treatment in patients with islet secretion disorders. First, single‐cell sequencing has demonstrated cellular heterogeneity within the islet [297] and even during diabetes progression [298, 299, 300]. However, its specific significance in the development, diagnosis, and treatment of diabetes remains to be elucidated. The intrinsic connections between various cells within the islet, including paracrine interactions, require further elucidation in the context of the progression of different types of diabetes. This understanding is essential for developing a comprehensive, multifaceted treatment strategy for diabetes. Second, studies have suggested that strategies to reconstitute a certain group of β cells may represent potential antidiabetic therapies [299]. Nevertheless, a substantial number of clinical studies remain necessary for its effective translational application. Furthermore, the discrepancies between mouse and human islets indicate that numerous findings from multiomics studies require further elucidation in the context of human islets. In addition, pancreatic islet transplantation represents a potential functional cure for diabetes [301, 302]. After transplantation, these cells have the potential to reverse diabetes in patients by restoring glycemic control. Nonetheless, the challenge of ensuring cell survival remains an urgent issue that requires resolution, which limits the widespread applicability of this procedure. The restricted availability of donor pancreases, along with the quantity and quality of the islets procured, are critical factors. Following transplantation, immune rejection adversely impacts islet survival, whereas prolonged use of antirejection medications detrimentally influences patients’ quality of life. These challenges have prompted the exploration of the intrinsic relationships among islet cells, the intricate pathogenesis of diabetes, and diverse alternative treatment modalities. Consequently, several cutting‐edge research initiatives are concentrated on this topic, encompassing areas from mechanistic exploration to clinical treatment (Figure 7).

### 6.1 Artificial Intelligence for Predicting Islet Hormone Dynamics

Recent advancements in artificial intelligence (AI), including image recognition, natural language processing (NLP) and computer vision, have shown particular promise for addressing key challenges in data analysis and monitoring [303]. In particular, in some instances, large language models (LLMs), such as Deepseek [304] and ChatGPT [305], have exhibited intelligence that goes beyond human capabilities. Fundamentally, diabetes and hormone secretion are often complex and multifactorial, making identifying effective methods for analysis and treatment difficult. The intricate mechanisms intrinsic to the islet, coupled with the complexity of drug development for disease treatment, present a multifaceted challenge involving multiple stages where setbacks can doom the entire process.

AI has already been implemented in pancreatic islet research. For example, using cryogenic electron microscopy alongside the AlphaFold protein structure prediction platform, the three‐dimensional spatial structure of SSTRs is continually being elucidated [306, 307, 308, 309, 310], which provides new knowledge on SSTR ligand recognition, the selectivity of SSTR isoforms, the structure of the SSTR‐G protein complex, and the activation process of SSTRs. The robust data processing capabilities of artificial intelligence effectively characterize its three‐dimensional structure. This detailed representation facilitates a more comprehensive exploration of potential mechanisms and clinical applications [311].

In addition to predicting three‐dimensional spatial structure, AI can also be utilized for the surveillance and analysis of clinical hormone levels. The regulation of hormones necessitates continuous feedback and adjustment mechanisms to prevent potential complications. For example, patients with diabetes frequently experience wide glycemic excursions, with common occurrences of both hypoglycemia and hyperglycemia, especially in patients with end‐stage kidney disease and poor adherence [312, 313]. Consequently, real‐time monitoring of blood hormone and glucose levels is essential. The implementation of continuous monitoring via artificial intelligence technology not only alleviates the inconvenience associated with traditional blood collection and monitoring methods but also facilitates ongoing analysis and prediction, enabling timely and automated interventions.

### 6.2 Gut Microbiome–Islet Axis

As previously mentioned, the gut–islet axis affects the regulation of hormone secretion. Importantly, the gut microbiota plays a significant role in this axis. Vatanen et al. reported the characterization of the early gut microbiome in connection with islet autoimmunity and T1D diagnosis [314], which suggested the protective effects of the microbiomes of children in early‐onset human T1D. Another study also demonstrated that early‐life microbial exposures determine sex hormone levels and modify progression to autoimmunity in the nonobese diabetic mouse model of T1D [315]. Transferring the gut microbiota from adult males to young females changed the recipient's microbiota, leading to increased testosterone levels, metabolomic alterations, decreased islet inflammation and autoantibody production, and strong protection against T1D. Therefore, for therapy, fecal microbiota transplantation (FMT) might halt the progression of human T1D. de Groot et al. conducted a randomized controlled trial to provide evidence for FMT [316]. Patients aged 18–30 years with recent‐onset T1D (less than 6 weeks) were randomly assigned to two groups. Over a 4‐month period, these groups underwent three FMTs, either autologous or allogeneic, from healthy donors. This study evaluated the preservation of C‐peptide release, glycemic control, fasting plasma metabolites, and alterations in the composition of the gut microbiota. They reported that FMT halted the decline in endogenous insulin production in recently diagnosed patients with T1D at 12 months after disease onset.

Therefore, further investigations of the role of the microbiota in the regulation of islet hormones to decelerate or reverse the progression of T1D by targeting the microbiota are becoming more prominent. For example, gut microbiota metabolites stimulate GLP‐1 secretion and influence its function and rhythm, whereas GLP‐1 impacts the gut microbiota through inflammatory response mechanisms [317]. From a therapeutic perspective, the administration of probiotics or prebiotics to modulate the gut microbiota and enhance GLP‐1 homeostasis represents a potential treatment strategy for diabetes.

### 6.3 Artificial Islets

Artificial islets are designed structures that mimic functional islets or islet organoids and were developed via biological and engineering methods. One strategy involves the use of biotechnological techniques to encapsulate pancreatic β cells for artificial pancreas transplantation. Alternatively, another approach focuses on the development of an artificial pancreas through the simulation of biological insulin release via computational algorithms. Generally, the latter artificial islet, made up of an insulin pump, a continuous glucose monitor, and a control algorithm, is automated for glucose‐responsive insulin delivery and mimics endogenous insulin release. The algorithm was programmed to minimize both high and low glucose concentrations and achieve improved glycemic control. Consequently, a diverse array of biological materials, synthetic chips, and other technological innovations have been employed in their development [318]. Hydrogels are extensively used in the creation of artificial islets because of their excellent biocompatibility and ease of handling [319]. For example, Delcassian et al. developed a dual‐function nanoparticle‐loaded hydrogel microcapsule [320]. This hydrogel microcapsule not only has the capacity to reestablish normoglycemia but also facilitates graft localization via magnetic resonance imaging. In particular, the artificial pancreas, developed by encapsulating β cells within a hydrogel matrix, demonstrates exceptional biocompatibility. Preclinical studies indicate that transplantation of this artificial pancreas does not elicit an inflammatory response [321]. Therefore, biocompatible polymers have been used to encapsulate islets, creating physical barriers that protect transplanted islets and prevent immune responses in recipients.

Recent advancements in 3D printing, biomaterials, and cell biology have led to the development of 3D bioprinting technology, which offers solutions for artificial islets. Liu et al. reported a custom‐designed coaxial printer for 3D printing of multicellular islet‐containing constructs [322]. 3D constructs can be fabricated with precise control over the arrangement of multiple cell types, and the viability of pancreatic islets is preserved after the 3D printing process. Chen et al. engineered a 3D‐printed encapsulation system incorporating β‐like cells and microvascular fragments to produce a retrievable microdevice with vascularized islets in vivo, which not only reduced the infiltration of immune cells but also showed excellent therapeutic efficacy and high safety [323]. Nevertheless, a solitary artificial islet comprising only β cells is inadequate to replicate the complex microenvironment and multifaceted functions of a natural pancreatic islet. Kim et al. [324] reported a bioprinted islet that presented structural and functional features resembling those of native islets. They used bioprinting‐based geometrical guidance to recreate the spatial pattern of islet peripheries and optimized the combination of pancreatic tissue‐specific ECM and basement membrane proteins, which improved stem cell‐derived islet functionality. Therefore, future advancements in tissue engineering techniques will prioritize the replication of the islet microenvironment to closely resemble that of natural islets, thereby facilitating the full expression of their physiological functions.

However, the lack of adequate nutrients and oxygen threatens the survival of artificial islets, requiring sophisticated alterations of biomaterials with specific chemical and physical properties. Moreover, the islet‐on‐a‐chip concept acts as a groundbreaking platform for islet studies, emphasizing the interaction between organs, which is consistent with the wider organ‐on‐a‐chip trend. In addition, AI also contributes to the conceptualization and design of artificial islets, whereas three‐dimensional printing technology facilitates the implementation of islet cell‐based transplantation therapies. Therefore, artificial islets have significant potential for future organoid research and diabetes therapy.

## 7 Conclusion

The intricate network of pancreatic islet hormones—insulin, glucagon, somatostatin, PP, and ghrelin—forms a highly coordinated system essential for metabolic homeostasis. The secretion and function of these hormones are regulated through a complex interplay of intrinsic nutrient sensing, paracrine and autocrine signaling, neural inputs, and gap junction‐mediated cell‐to‐cell communication, with novel mechanisms currently being discovered. Disruptions in this finely tuned network underlie the pathogenesis of diabetes, where imbalances in hormone secretion led to hyperglycemia, impaired insulin action, and dysregulated counterregulatory responses. Understanding the secretion mechanisms and interplay between these hormones provides critical insights into both normal physiology and the dysregulation observed in diabetes.

Recent advances in our understanding of islet biology and emerging technologies have further elucidated the regulatory mechanisms of hormone secretion in islet cells and their relevance to diabetes management, revealing new therapeutic opportunities. Emerging strategies, ranging from glucose‐responsive insulin and multireceptor modulators to CRISPR‐based gene correction, microencapsulated islet transplantation, ECM‐driven pancreas bioengineering, and bioelectronic modulation, aim to restore physiological hormone dynamics rather than merely compensating for deficiencies. The development of targeted therapies that address both insulin deficiency and glucagon excess while preserving intraislet crosstalk holds particular promise for achieving durable glycemic control.

The integration of single‐cell omics, gene editing, and advanced biomaterials will further refine our ability to modulate islet hormone function with precision. However, challenges remain in translating these innovations into clinical practice, including ensuring long‐term safety, overcoming immune rejection, and addressing the functional maturation of engineered cells. By embracing a multidisciplinary approach that bridges immunology, bioengineering, and neuroendocrinology, future therapies may ultimately achieve the goal of restoring true metabolic equilibrium in diabetes, moving beyond symptomatic management toward curative strategies. The continued exploration of islet hormone networks will undoubtedly yield transformative insights, offering hope for millions affected by diabetes worldwide.

## Author Contributions

Jinfang Ma, Mao Li, Xing Huang, and Zhenyu Duan reviewed the literature and drafted the manuscript. Lingxiao Yang, Qingxiang Xie, Rongping Fan, Xi Lu, and Nanwei Tong reviewed the relevant literature. Jinfang Ma, Mao Li, Xing Huang, Nanwei Tong, and Zhenyu Duan designed and reviewed the manuscript. All the authors have read and approved the final version of the manuscript.

## Conflicts of Interest

The authors declare no conflicts of interest.

## Ethics Statement

The authors have nothing to report.

## Data Availability

The authors have nothing to report.
